# Variation in Chemical Composition and Biological Activities of *Flos Chrysanthemi indici* Essential Oil under Different Extraction Methods

**DOI:** 10.3390/biom9100518

**Published:** 2019-09-21

**Authors:** Chang-Liang Jing, Rui-Huan Huang, Yan Su, Yi-Qiang Li, Cheng-Sheng Zhang

**Affiliations:** 1 Ocean Agriculture Research Center, Tobacco Research Institute of Chinese Academy of Agricultural Sciences, Qingdao 266101, China; jingchangliang@caas.cn (C.-L.J.); 18779368572@163.com (R.-H.H.); 2 Chongqing key laboratory of traditional Chinese medicine resources, Chongqing Academy of Chinese Materia Medica, Chongqing 400065, China; cqsuyan@126.com

**Keywords:** *Flos Chrysanthemi indici*, essential oil, extraction method, chemical composition, biological activity

## Abstract

*Flos Chrysanthemi indici*, an important medicinal and aromatic plant in China, is considered to have many different preservative and pharmacological properties. Considering the capability of essential oils (EOs), the present study is conducted to compare different extraction methods in order to improve yield and biological activities. Hydro-distillation (HD), steam-distillation (SD), solvent-free microwave extraction (SFME), and supercritical fluid extraction (SFE) are employed to prepare EOs from *Flos Chrysanthemi indici*. A total of 71 compounds are assigned by gas chromatography/mass spectrometry (GC–MS) in comparison with retention indices. These include 32 (HD), 16 (SD), 31 (SFME) and 38 (SFE) compounds. Major constituents of EOs differ according to the extraction methods were heptenol, tricosane, camphor, borneol, and eucalyptol. EOs extracted by SFME exhibit higher antioxidant activity. All EOs show varying degrees of antimicrobial activity, with minimum inhibitory concentration (MIC) ranging from 0.0625 to 0.125 mg/mL and SFME and SFE prove to be efficient extraction methods. EOs alter the hyphal morphology of *Alternaria alternata*, with visible bumps forming on the mycelium. Overall, these results indicate that the extraction method can significantly influence the composition and biological activity of EOs and SFME and SFE are outstanding methods to extract EOs with high yield and antimicrobial activity.

## 1. Introduction

*Flos Chrysanthemi indici* derived from *Chrysanthemum indicum* L. is listed in the Chinese Pharmacopoeia (2005) for medical use and distributed across most parts of China. Studies show that *Flos Chrysanthemi indici* has anti-inflammatory and antibacterial activities and is effective in neutralizing infectious diseases [1,2]. Cineole, borneol, and bornyl acetate are the principal constituents of essential oils (EOs) contributing to their medicinal properties [3].

EOs can be obtained from different plants by many methods including hydro-distillation (HD), steam-distillation SD, solvent-free microwave extraction SFME, and supercritical fluid extraction SFE [4,5,6]. Previous studies showed that extraction methods could affect the composition of EOs and further result in different bioactivities [7]. Currently, HD is the principal approach for the extraction of essential oil from *Flos Chrysanthemi indici*. This approach suffers some disadvantages such as time-consumption, the requirement of extensive energy consumption, and low efficiency. However, SFME and SFE have emerged as efficient alternative methods among the others for essential oil separation from plant materials [8,9].

Diseases that are caused by microorganisms lead to large postharvest losses. Fruits and vegetables can be attacked by microorganisms during the production and postharvest stages, leading to significant economic losses for the food industry [10]. In the case of rice, fruits, and vegetables, the most common diseases were caused by *Xanthomonas oryzae* pv. *oryzae*, *Ralstonia solanacearum*, *Pseudomonas syringae* pv. *lachrymans*, *Acidovorax citrulli*, *Rhizoctonia solani*, and *Erwinia carotovora* [11,12]. Chemical agents are typically used to inhibit the growth of contaminating microorganisms. However, due to growing concerns over the safety of foods containing chemical additives, natural antibacterial products for fruit and vegetable preservation are attracting increasing attention. EOs are naturally synthesized in aromatic plants as secondary metabolites [13] and exhibit potent suppressive activity against bacteria, molds, and yeasts, and have therefore been used in food preservation and natural remedies [14,15,16]. EOs have the added advantages of being volatile, eco-friendly, and biodegradable [17,18], which makes them acceptable to consumers [19].

To the best of our knowledge, application of SFME and SFE to obtain essential oil from *Flos Chrysanthemi indici* has not been previously reported. There is also no reported research to compare the chemical composition and biological activities, including the antimicrobial and antioxidant properties of essential oils, obtained from different extraction methods. To address these issues, the present study investigates the yield, chemical composition, and biological activities of EOs extracted from *Flos Chrysanthemi indici* by four different methods, including HD, SD, SFME and SFE, analyzed by gas chromatography/mass spectrometry (GC–MS). In order to identify the most effective extraction method in terms of their activities, we investigated by 2,2-diphenyl-1-picryl-hydrazyl-hydrate (DPPH), 2,2’-Azinobis-(3-ethylbenzthiazoline-6-sulphonate) (ABTS) assays for antioxidant activities and minimum inhibition concentration for antimicrobial activities. Moreover, the results of the present study might be of help to finding high quality essential oil with antioxidant and antimicrobial activities. 

## 2. Materials and Methods

### 2.1. Plant Material and Chemicals

*Flos Chrysanthemi indici* were identified by Prof. Chengsheng Zhang [20] at the sampling sites. Fresh flowers were collected from coastal shoals in Shandong province of China in October 2017. The materials were dried at room temperature and mechanically grounded in a laboratory mill to a homogenous powder and then sieved through a 0.45 mm sifter. All chemicals and solvents used in this study were of analytical grade.

### 2.2. Extraction Method

#### 2.2.1. Hydro-Distillation (HD)

*Flos Chrysanthemi indici* powder was weighted (100 g) and placed in a round bottom flask with 1000 mL distilled water as extraction solvent; the material-water mixture was refluxed for about 240 min (until no more extracts was obtained), during which the extract was collected in the side arm of the Clevenger-type apparatus. The product was dried with anhydrous sodium sulfate and stored at 4 °C until analysis. The process was performed three times. Yield percentage was calculated using the volume (mL) of EO per 100 g of dried *Flos Chrysanthemi indici* powder.

#### 2.2.2. Steam-Distillation Method (SD)

Glassware and operating conditions were the same as those used in HD. The vapor produced by the steam generator passed through the EO-rich plant material before condensing in a receiving Clevenger-type apparatus [21]. The product was dried over anhydrous sulfate and stored at 4 °C until analysis. The extraction was performed three times.

#### 2.2.3. Solvent-Free Microwave Extraction (SFME)

Solvent-free microwave extraction was carried out using Uwave-200, Sineo Microwave (Chemistry Technology Co., LTD, Shanghai, China). This instrument was equipped with an infrared temperature sensor, an electromagnetic stirrer, a time controller and a circulating water-cooling system. The extraction procedure followed the method previously described [22] with some modifications. Briefly, 100 g of *Flos Chrysanthemi indici* were placed in a 1 L flask and soaked for 30 min. The flask was placed inside the microwave oven and a condenser was fitted to the top (outside the oven) to collect extracted EO. The vapor produced by microwaves passed through the materials before condensing in a receiving Clevenger-type apparatus. The microwave oven was operated at 800 W for a period of 30 min, during which EO was collected from the top of the Clevenger apparatus. The product was dried over anhydrous sodium sulfate, weighed, and stored in dark brown vials at 4 °C until use. The extraction was performed three times.

#### 2.2.4. Supercritical Fluid Extraction (SFE)

Supercritical fluid extractions were performed on a laboratory scale supercritical fluid extraction system (Waters, SFE-500M1-2-C10, USA). All extractions were performed with supercritical CO_2_ in dynamic mode. The ground sample (100 mg) was loaded into 50 mL high pressure vessel connected in series and the CO_2_ was pressurized using a high pressure pump and then charged into the vessel at the rate of 20 g/min to maintain the desired pressure of 20 MPa during the cycle. The supercritical CO_2_ containing the extract was then passed through a temperature-controlled micrometer valve and was expanded to ambient pressure. The volume of EOs obtained were measured and filled up to 10 mL with hexane and stored in dark brown vials at 4 °C until use. The sample was extracted in triplicate.

### 2.3. Gas Chromatography-Mass Spectrometry (GC–MS) Analysis

The gas chromatography-mass spectrometry (GC–MS) analysis of the EOs was performed using an Agilent GC–MSD system as previously described [23]. The system consisted of an Agilent Technologies 7890A gas chromatograph (Santa Clara, CA, USA) equipped with a Mars 6100 ion trap mass detector (USA). The separation was done on a DB-5MS capillary column (30 m × 0.25 mm id × 0.25 µm, Bellefonte, PA, USA). The vector gas was helium and the flow rate was 1.0 mL/min, injection volume was 1 µL, 1:20 split ratio; injection temperature was 250 °C, oven temperature was held at 40 °C for 5 min and then increased to 250 °C by 20 °C/min and held at 250 °C for 5 min; 70 eV ionization energy; 30–300 µscan range; quadrupole temperature at 150 °C, ion source temperature at 230 °C. The identity of each component was assigned by comparison of their retention time and mass spectrum with that of a standard from the NIST08 database provided with the GC–MS system software. The relative contents of the components were calculated by comparing its GC peak area to the total areas that are summed from all detected peaks. 

### 2.4. Antioxidant Capacity Evaluation

#### 2.4.1. DPPH Radical-Scavenging Activity Assay

The DPPH radical scavenging activity was measured based on the method described previously [24] with some modifications. In brief, 50 µL of EOs at different concentrations were mixed with 2 mL of DPPH (60 µM) methanol solution. The EOs were mixed well with the solution. After 30 min sealed incubation at room temperature in darkness, absorbance was recorded at 517 nm. The DPPH antioxidant activity was calculated using the following formula: DPPH scavenging activity (%) = [1 − (A_sample_ − A_sample blank_)/A_control_] × 100, where A_sample_ represents absorbance of the ethanol solution of DPPH with tested samples; A_sample_ blank represents absorbance of the extracts without the ethanol solution of DPPH and A_control_ was prepared without sample (which was replaced by distilled water) and the sample concentration providing 50% inhibition (IC_50_) was calculated by plotting inhibition percentages against concentration of the sample. The results were reported as the average of three replicates. 

#### 2.4.2. ABTS Radical Scavenging Activity

The ABTS radical scavenging activity of extracts was determined using the method described previously [25] with some modifications. The ABTS+ solution was prepared from the reaction of equal volumes of 2.45 mM potassium persulfate and 7 mM ABTS in a dark place at ambient temperature for 16 h. The ABTS+ solution was adjusted to the absorbance of 0.700 ± 0.02 at 734 nm with ethanol. An aliquot of 0.5 mL EOs (2 mg/mL) was mixed with 1.5 mL ABTS+ solution and sealed and incubated at room temperature for 6 min, the absorbance was measured at 734 nm. The EOs were mixed well with the solution. The ABTS scavenging activity of extracts was calculated as follows: ABTS scavenging activity (%) = [(A_control_ − A_sample_)/A_control_] × 100, where A_control_ represented absorbance without sample and A_sample_ represented absorbance with sample. The results were reported as the average of three replicates. 

### 2.5. Evaluation of Antibacterial and Antifungal Activities

*Ralstonia solanacearum* (tobacco bacterial wilt) were obtained from the Tobacco Research Institute (Qingdao, China). *Acidovorax citrulli* (bacterial fruit spot), *Pseudomonas syringae* pv. *lachrymans* (angular leaf spots in cucumbers), *Erwinia carotovora* (Chinese cabbage soft rot), *Xanthomonas oryzae* pv. *oryzae* and *Rhizoctonia solani* were provided by the Plant Protection Research Institute of CAAS. 

The antibacterial activity of EOs obtained by different method against six plant pathogenic bacteria including *Xanthomonas oryzae* pv. *oryzae*, *Ralstonia solanacearum*, *Pseudomonas syringae* pv. *lachrymans*, *Acidovorax citrulli*, *Rhizoctonia solani*, and *Erwinia carotovora* as tested by disk diffusion method. *Ralstonia solanacearum* (tobacco bacterial wilt) were obtained from the Tobacco Research Institute. *Acidovorax citrulli* (bacterial fruit spot), *Pseudomonas syringae* pv. *Lachrymans* (angular leaf spots in cucumbers), *Erwinia carotovora* (Chinese cabbage soft rot), *Xanthomonas oryzae* pv. *Oryzae* and *Rhizoctonia solani* were provided by the Plant Protection Research Institute of CAAS. 

Briefly, the six plant bacteria were cultured in Luria–Bertani (LB) broth at 37 °C overnight and then adjusted to a concentration of 10^6^ CFU/mL. One hundred microliters of bacterial suspensions were spread on petri dishes containing 10 mL LB broth with agar and the petri dishes were divided into four parts. Sterile filter paper disks (6 mm in diameter) impregnated with 10 µL of EO, dimethylsulfoxide (DMSO) (negative control), and aqueous streptomycin sulfate solutions (10 mg/mL) (positive control) were placed on the cultured plates. The treated petri dishes were sealed and incubated at 37 °C for 24 h.

Antifungal activity of EOs was tested using the method described previously [26] with some modifications. A mycelial agar disk (diameter = 7 mm) isolated from a 7-day culture was placed at the center of a petri dish containing 15 mL Potato-Dextrose-Agar (PDA) medium. EOs were dissolved in DMSO to a final concentration of 1.0 mg/mL, and 15 µL of EOs solutions were added to each of the symmetrically holes (diameter = 6 mm) on the dishes and then incubated at 28 °C for five days. Dishes with added DMSO served as a negative control and streptomycin sulfate solution (10 mg/mL) as a positive control. Antifungal activity was evaluated by observing the inhibition zones after five days.

### 2.6. Determination of Minimum Inhibitory Concentration (MIC)

The minimal inhibitory concentration (MIC) was determined by the microdilution method as described previously [27]. Subsequent serial dilutions were performed on sterile 96-well microplates. EO was first diluted to the highest concentration of 0.5 mg/mL and serially diluted (two fold dilutions) with DMSO to a final concentration of 0.03125 mg/mL. The bacterial suspension was adjusted to 10^6^ CFU/mL using a 0.5 McFarland turbidity standard. Finally, 100 µL of standard bacterial suspension were added to each well containing different concentrations of EO, and the plates were incubated at 37 °C for 24 h. The MIC of the EO was determined as the lowest concentration that visibly inhibited bacterial growth.

The MIC of EO against a plant pathogenic fungal strain was determined as previously described [28] with some modifications. The strain was grown in potato dextrose water (PDW) cultures at 28 °C for 72–120 h until the plate was covered. Fungal samples were harvested using a 5 mm sterilized puncher along the edge of the colonies and added to the center of plates containing different concentrations of EOs. The plates were cultured on a rotary shaker at 150 rpm for 20 s and incubated at 28 °C for 48 h. Each treatment was prepared in triplicate. The MIC values were determined as the lowest concentration required to inhibit fungal growth.

### 2.7. Scanning Electron Microscopy (SEM)

To evaluate morphological changes in fungi treated with EO, SEM analysis was performed according to previously described methods [23] with some modifications. About five 10 mm mycelium segments were excised from the cultured plates with EOs and washed 3 times with normal saline. The segments were fixed with 2.5% (*v*/*v*) glutaraldehyde (4 °C, 2 h) and washed with 0.1 M PBS (pH 7.0). Then the samples were dehydrated in a graded series of ethanol (30%, 50%, 70%, and 100%) for 30 min each, dried in liquid CO_2_, and viewed with a scanning electron microscope (Hitachi S3400N, Hitachi Science Systems, Ltd., Ibaraki, Japan) operated at 20 kV at a magnification of 100,000×.

### 2.8. Data Analysis

Statistical analysis was performed using Statistical Analysis System version 9.2 software (SAS Institute, Cary, NC, USA.). All analyses were performed in triplicate and the data were reported as means ± standard deviation (SD) of three samples. *p* < 0.05 was considered statistically significant. 

## 3. Results and Discussion

### 3.1. Effect of Extraction Method on EO Yield

**Figure 1:**
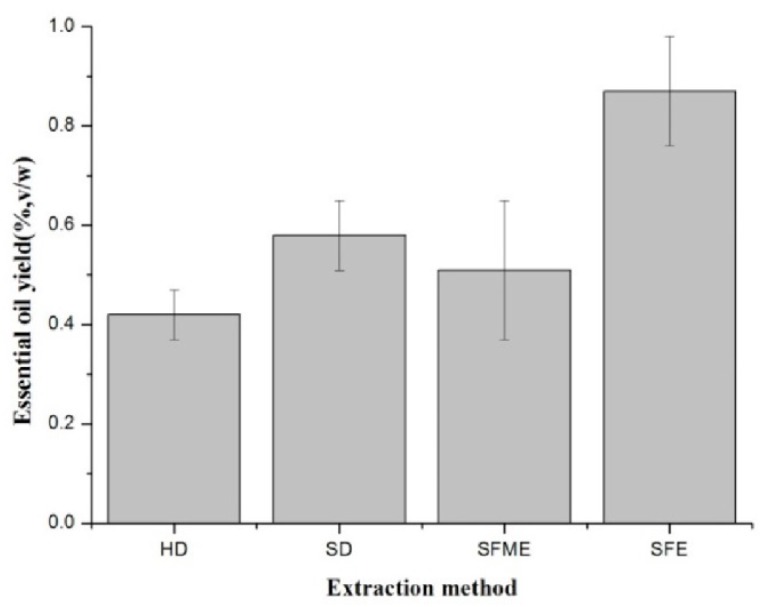
Changes in essential oil yields (%, *v*/*w*) of *Flos Chrysanthemi indici* under different extraction methods.

The results illustrated in Figure 1 showed that *Flos Chrysanthemi indici* EO yields were affected by extraction method. It was reported that SFME and SFE are more eco-friendly and efficient technologies for extracting EOs than HD [29,30]. In this study, the highest yield was obtained by SFE (0.87 ± 0.11% *v*/*w*, dry weight basis) and the lowest by HD (0.42 ± 0.05%, *v*/*w*). This is consistent with a previous report that SFE was the optimal process for obtaining a high yield of EOs from clove buds [31]. Supercritical fluid possesses gas-like properties of diffusion, surface tension, liquid-like density, viscosity, and solvation power. Thus, the higher yield may be due to the favorable transports properties of fluids near their critical points allow deeper penetration into matrix and more efficient and faster extraction [8,32].

### 3.2. Effect of Extraction method on EOs Composition

**Figure 2:**
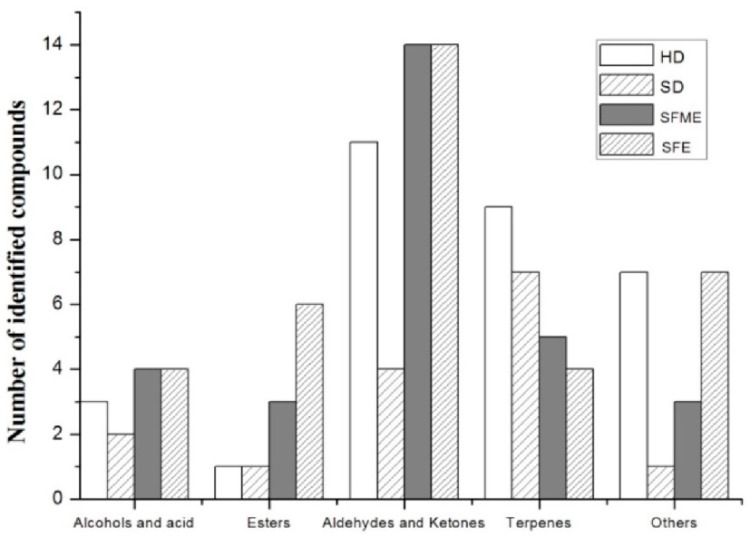
Numbers of the classes of compounds in extracts obtained by different extraction methods.

In total, 60 compounds were identified by GC–MS analysis which were obtained by four extraction methods, including eight alcohols and acid compounds, eight esters, five aldehydes and ketones, 19 aliphatic hydrocarbons, 11 monoterpenes and sesquiterpenes and nine other compounds. The categorization of assigned compounds in various extracts is shown in Figure 2. It can be observed that the EOs obtained by SFE has a higher content of alcohols and esters and the EOs obtained by HD contains higher terpenes. 

The main differences among the four extraction methods are composition of individual groups. Comparative composition of the EOs obtained from different extraction method are shown in Table 1, 35 compounds were identified in SFE extracts, and 31 compounds in HD extracts, 15 compounds in SD extracts, 29 compounds in SFME extracts, respectively. Only five compounds were identified in all different EOs (Bornyl acetate, 1,7,7-Trimethylbicyclo[2.2.1]hept-5-en-2-one, Eucalyptol, Camphor and isobenzofuranone). The main compounds detected of individual extraction method were heptenol, borneol, eucalyptol, camphor, and caryophyllene oxide. Other detected compounds were present in concentrations less than 2%.

From the extraction yield and the total numbers of assigned compounds, SFE was a more effective method. Nevertheless, borneol and caryophyllene oxide were not detected in the SFE extract. If we consider the main compounds content, SFME was a more effective technique and contains the highest amount of heptenol, borneol, caryophyllene oxide, and trans-β-Farnesene. The SFME and SFE produced essential oils with higher quantities of valuable oxygenated compounds. This may be due to the reduced heating time required, which partially prevented decomposition oxygenated compounds. Comparatively, it could be noticed that camphor (relative content 39.71 ± 2.89%) and eucalyptol (relative content 11.38 ± 2.47%) were found in the highest quantities in the SD extract and the two compounds are regarded as parameters for assessing the quality of *Flos Chrysanthemi indici* [33]. Camphor has various biological effects including antimicrobial, insecticidal, and antiviral activities [34]. Camphor was also reported in Chuzhou *chrysanthemum* and *Chrysanthemum boreale Makino* essential oil and showed the highest component at vegetative stage in *Chrysanthemum boreale Makino* [35,36]. 

### 3.3. Bioactivity of EOs

#### 3.3.1. Antioxidant Activities

Antioxidant activity is a critical indicator to reveal the bioactivity of natural products. It is necessary to use different antioxidant assays to estimate antioxidant activity of a product. In this study, we employed two assays (DPPH radical scavenging assay and ABTS scavenging assay) to determine the antioxidant activity of the four EOs. Ascorbic acid was used as positive controls. The results are given in Table 2.

Regardless of the extraction method and antioxidant assays, all studied EOs possessed significantly weaker free radical scavenging activity than the synthetic antioxidant ascorbic acid. The radical scavenging ability of examined EOs was higher in the DPPH assay than in the ABTS assay. EOs extracted by HD and SD possessed weaker free radical scavenging power than that by SFE and SFME by DPPH assay and EOs extracted by SD and SFME possessed higher antioxidant than that by HD and SFE. These differences may be explained by the complex constituents which are considered to be effective antioxidant compounds. A number of reports are available in the literature that the antioxidant activities of essential oils may act synergistically and the activity may be higher than a single compound [37].

#### 3.3.2. Antibacterial Activity

**Figure 3:**
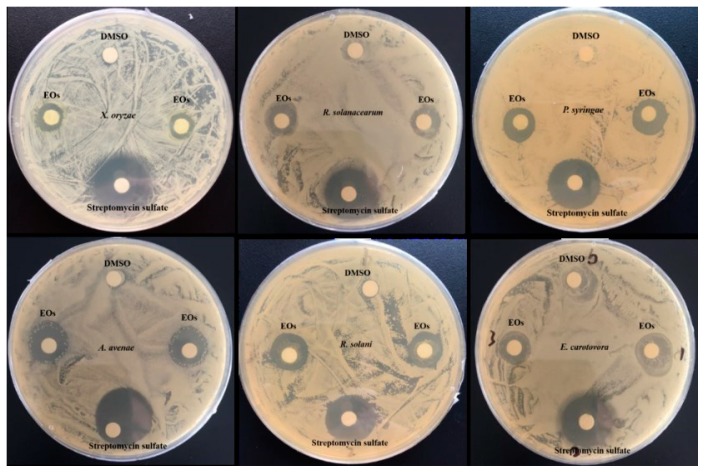
Antibacterial activity of EOs extracted from *Flos Chrysanthemi Indici* evaluated by disk diffusion method.

The antibacterial activities of the obtained EOs are presented in Table 3. This is the first report describing the antibacterial activities of EOs extracted from *Flos Chrysanthemi indici* by different methods. We evaluated the antibacterial activity of EOs against six plant pathogenic bacteria including *X. oryzae, R. solanacearum, P. syringae, A. avenae, R. solani, and E. carotovora* and found that the growth of all six strains was inhibited in the presence of EOs (Figure 3). 

We compared the antibacterial activities of EOs obtained by different extraction methods based on the MIC against the six bacterial strains. All of the EOs showed varying degrees of antibacterial activity, with MIC ranging from 0.0625–0.125 mg/mL (Table 3). EOs extracted by the four extraction methods had the same MIC against *P. syringae* and *R. solani*. EOs extracted by SFE showed the lowest MIC against *R. solanacearum* (MIC of 62.5 µg/mL), whereas those extracted by HD and SFME showed the lowest MIC against *A. avenae* (MIC of 62.5 µg/mL). They were all higher than the positive control, with MIC 17.15 and 1.953 µg/L, respectively. Compared with other EOs, Mohamed et al. reported that EOs from *Cupressus sempervirens*, *Lantana camara* and *Corymbia citriodora* showed antibacterial effects against *Ralstonia solanacearum* from potato with MIC ranged from 8–5000 µg/mL [38]. Sabir et al. reported that EOs from local plants showed antibacterial against *Pseudomonas syringae* with MIC ranged from 31.25–500 µg/mL [39]. These observations could be explained by differences in the chemical composition of obtained EOs. All EOs obtained by the four methods were rich in active camphor (39.71–7.14%), which could account for the antibacterial activity of *Flos Chrysanthemi indici* EOs.

#### 3.3.3. Antifungal Activity

Several EOs have been shown to be effective in the control of postharvest fungal pathogens, including *A. alternate.* We also tested the antifungal activity of EOs from *Flos Chrysanthemi indici* against *A. alternate* and found that fungal growth was significantly inhibited by SFME obtained EOs with a MIC of 0.625 mg/mL. In prior reports, Castro et al. [40] who tested six kinds of EOs against *A. alternata* in dragon fruit*,* found that the MIC value ranged from 250–1000 µg/mL. The MIC of EOs of *Flos Chrysanthemi indici* in this study were generally lower than some of the EOs from other plants, such as *Cymbopogon flexuosus*, *Eucalyptus globulus* and *Rosmarinus officinalis* [40].

**Figure 4:**
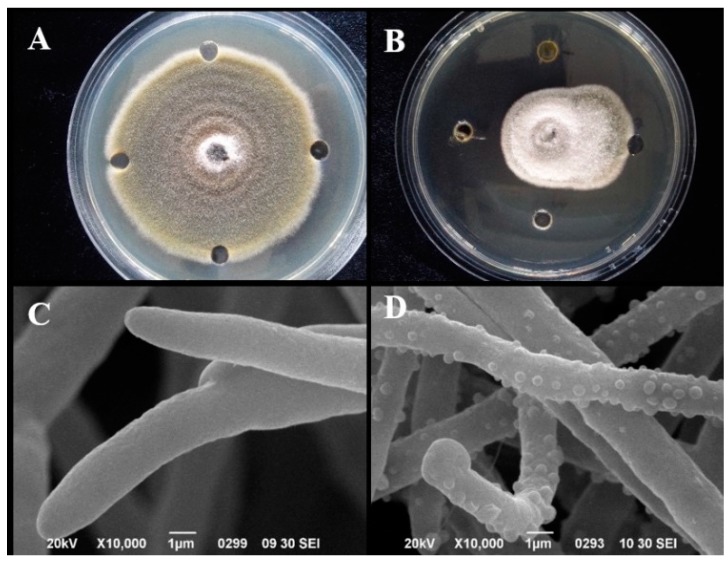
Antifungal bioassay against *A. alternata* and scanning electron micrographs (10,000×) of the morphology of *A. alternata* with and without EO treatment. (**A**) Control group; (**B**) *A. alternate* treated with EOs; (**C**) mycelium of untreated culture (control); (**D**) mycelium of *A. alternata* treated with EOs at MIC.

To investigate the mechanism associated with the antifungal activity of the EO, we examined the cell membrane structure of spores treated with the MIC by SEM (Figure 4C,D). In the control group, the mycelium retained a normal morphology with intact cell wall and membrane (Figure 4C). However, the mycelium of *A. alternata* treated with 0.625 mg/mL EO showed significant morphological changes, including bumps on the surface (Figure 4D). This differs from the reported effects of other EOs on this strain [23].

## 4. Conclusions

This study presents results of the analysis of EOs obtained from *Flos Chrysanthemi indici* by using various extraction methods. We found that EOs yield, chemical composition, and bioactivities varied according to the extraction method. The extraction yield of SFE was higher than other methods. The SFME and SFE method produced EOs with higher quantities of valuable oxygenated while HD method produced higher quantities terpenes. Bioassay indicated that EOs contain compounds with antioxidant and antimicrobial activities against several pathogenic strains and varied according to different extraction methods. Nonetheless, it was detected that EOs of *Flos Chrysanthemi indici* extracted by any one of the four methods inhibited the growth of these plant pathogens and SFME and SFE proved to be higher in bioactivity. These results can provide essential information for the application of EOs obtained from different extraction methods in food, beverage and pharmaceutical industries. 

## Figures and Tables

**Table 1:** Volatile compounds identified in extracts of *Flos Chrysanthemi indici* prepared by different extraction methods.

No.	Name of Compound	Molecular Weight	CAS Number	Extraction Method ^a^							
				HD		SD		SFME		SFE	
				RT ^b^	%A ^c^	RT	%A	RT	%A	RT	%A
1	Camphene	136.125	000079-92-5	9.642	0.42 ± 0.05	n.i	n.i	n.i	n.i	n.i	n.i
2	Eucalyptol	154.136	000470-82-6	11.388	3.59 ± 1.64	11.418	11.38 ± 2.47	11.38	0.69 ± 0.04	11.38	0.95 ± 0.96
3	1,4-Cyclohexadiene, 1-methyl-4-(1-methylethyl)-	136.125	000099-85-4	11.862	0.22 ± 0.05	11.862	0.28 ± 0.29	n.i	n.i	n.i	n.i
4	Methyl ethyl cyclopentene	110.11	019780-56-4	n.i	n.i	n.i	n.i	12.75	0.66 ± 0.21	12.746	0.66 ± 0.78
5	(*Z*,*Z*)-3,5-Octadiene	110.11	007348-80-3	12.754	1.45 ± 2.28	12.767	2.40 ± 0.47	n.i	n.i	n.i	n.i
6	lsobenzofuranone	182.094	054346-06-4	12.923	1.32 ± 0.47	12.936	1.97 ± 2.25	12.919	0.86 ± 0.98	12.923	0.57 ± 0.41
7	(*E*)-2-Hexen-4-yn-1-ol	96.058	053497-80-6	13.718	2.34 ± 1.12	n.i	n.i	n.i	n.i	n.i	n.i
8	1,7-Octadiene, 3,6-dimethylene	134.11	003382-59-0	n.i	n.i	n.i	n.i	13.722	2.99 ± 0.74	n.i	n.i
9	4-Ethyl-2-hexynal	124.089	071932-97-3	n.i	n.i	13.743	2.30 ± 0.87	n.i	n.i	n.i	n.i
10	endo-Borneol	154.136	000507-70-0	n.i	n.i	n.i	n.i	n.i	n.i	13.866	1.55 ± 0.06
11	Borneol	154.136	010385-78-1	13.909	7.07 ± 1.17	13.925	5.22 ± 2.74	13.913	8.01 ± 2.44	n.i	n.i
12	4-methyl-1-(1-methylethyl)-3-Cyclohexen-1-ol	154.136	000562-74-3	n.i	n.i	14.018	1.20 ± 0.24	n.i	n.i	n.i	n.i
13	1-butenylidene-Cyclohexane	136.125	036144-40-8	14.234	1.05 ± 0.25	n.i	n.i	14.234	0.96 ± 0.47	n.i	n.i
14	heptenol	194.131	1000195-66-0	14.746	17.99±1.25	n.i	n.i	14.746	16.66±2.31	14.716	6.93± 0.09
15	tert-butyl-Benzene	134.11	000098-06-6	n.i	n.i	15.13	24.12±2.36	14.746	0.15 ± 0.47	n.i	n.i
16	Bicyclo[3.1.1]hept-2-en-6-ol, 2,7,7-trimethyl-, acetate, [1S-(1. 1α,5α,6β)]-	194.131	050764-55-1	n.i	n.i	n.i	n.i	15.122	1.71 ± 1.21	n.i	n.i
17	Bornyl acetate	196.146	000076-49-3	15.545	2.06 ± 0.04	15.558	3.15 ± 2.14	15.545	1.33 ± 0.05	15.537	1.51 ± 0.23
18	Cosmene	134.11	000460-01-5	16.074	0.28 ± 0.06	16.078	0.26 ± 0.12	n.i	n.i	n.i	n.i
19	Caryophyllene	204.188	000087-44-5	n.i	n.i	17.473	0.43 ± 0.25	n.i	n.i	17.469	0.31 ± 0.01
20	Isocaryophyllene	204.188	000118-65-0	n.i	n.i	n.i	n.i	n.i	n.i	17.765	0.68 ± 0.99
21	a-Gurjunene	204.188	000489-40-7	18.137	0.27 ± 0.01	n.i	n.i	18.137	0.28 ± 0.04	n.i	n.i
22	Curcumene	202.172	000644-30-4	18.179	0.58 ± 0.11	n.i	n.i	18.184	0.84 ± 0.31	18.175	0.33 ± 0.11
23	Naphthalene	204.188	000473-13-2	18.37	1.28 ± 0.25	n.i	n.i	18.441	3.26 ± 1.02	18.366	0.21 ± 0.14
24	Spathulenol	204.188	025246-27-9	n.i	n.i	n.i	n.i	18.37	1.12 ± 0.08	n.i	n.i
25	1,1,7-Trimethyl-4-methylenedec ahydro-1*H*-cyclopropa[e]azulene	204.188	025246-27-9	18.37	0.53 ± 0.25	n.i	n.i	18.37	1.49 ± 0.79	n.i	n.i
26	trans-β-Farnesene	204.188	018794-84-8	18.251	2.66 ± 0.17	18.725	0.33 ± 0.45	20.213	5.07 ± 2.14	n.i	n.i
27	(*Z*)-3-Undecen-1-yne	150.141	074744-32-4	n.i	n.i	n.i	n.i	n.i	n.i	19.528	1.23 ± 0.28
28	Caryophyllene oxide	220.183	001139-30-6	19.541	4.23 ± 1.11	19.537	1.09 ± 0.52	19.554	7.33 ± 1.28	n.i	n.i
29	(*E*,*Z*)-α-Famesene	204.188	1000293-03-2	n.i	n.i	n.i	n.i	20.213	1.0 ± 0.04	n.i	n.i
30	1*R*,3*Z*,9*S*-4,11,11-Trimethyl-8-methylenebicyclo [7.2.0] undec-3-ene	204.188	1000140-07-3	20.416	1.35 ± 0.28	n.i	n.i	n.i	n.i	n.i	n.i
31	*cis*-Z-α-Bisabolene epoxide	220.183	1000131-71-2	20.501	0.49 ± 0.74	n.i	n.i	n.i	n.i	20.818	0.39 ± 0.08
32	*trans*-α-Bergamotene	204.188	013474-59-4	20.97	3.76 ± 0.45	n.i	n.i	n.i	n.i	n.i	n.i
33	2,6-dimethyl-6-(4-methyl-3-pentenyl)-Bicyclo [3.1.1] hept-2-ene	204.188	017699-05-7	n.i	n.i	n.i	n.i	20.974	4.98 ± 2.47	n.i	n.i
34	1,3-Bis-(2-cyclopropyl, 2-methylcyclopropyl)-but-2-en-1-one	258.198	1000222-08-6	n.i	n.i	n.i	n.i	n.i	n.i	20.975	1.31± 0.04
35	1,7,7-Trimethylbicyclo [2.2.1] hept-5-en-2-one	150.104	022516-10-5	21.288	5.89 ± 0.87	21.266	0.67 ± 0.12	21.296	8.18 ± 2.14	21.262	3.90 ± 1.25
36	Camphor	150.104	022516-10-5	21.288	23.53±5.21	21.266	39.71±2.89	21.296	13.51±1.57	21.262	7.14 ± 2.36
37	Naphthalenone	218.167	091416-23-8	21.381	1.59 ± 0.21	n.i	n.i		1.34 ± 0.51	n.i	n.i
38	6,6-Dimethyl-2-vinylidenebicyclo [3.1.1] heptane	148.125	039021-75-5	n.i	n.i	n.i	n.i	21.389	2.31 ± 0.58	n.i	n.i
39	5-benzyloxy-Pent-1-yne	174.104	057618-47-0	21.609	1.27 ± 0.28	n.i	n.i	n.i	n.i	n.i	n.i
40	octahydro-1,4,9,9-tetramethyl-1*H* -3a, 7-Methanoazulene	206.203	025491-20-7	21.93	0.34 ± 0.14	n.i	n.i	21.93	1.05 ± 0.43	21.93	0.22 ± 0.08
41	1,5-Heptadiyne	92.063	000764-56-7	n.i	n.i	n.i	n.i	n.i	n.i	22.07	1.06 ± 0.41
42	Phenylethyne	102.047	000536-74-3	n.i	n.i	n.i	n.i	n.i	n.i	23.152	0.25 ± 0.09
43	2,3-dihydroxy-1*H*-Cyclopenta[b] quinoxalin-1-one	214.038	023774-23-4	23.512	1.34 ± 0.96	n.i	n.i	23.512	4.76 ± 1.12	23.512	7.63 ± 1.25
44	2,3,4,5-tetramethyl-Tricyclo [3.2.1.02,7] oct-3-ene	162.141	062338-44-7	n.i	n.i	n.i	n.i	n.i	n.i	23.668	7.62 ± 1.47
45	Octacosane	394.454	000630-02-4	n.i	n.i	n.i	n.i	n.i	n.i	24.789	1.81 ± 0.21
46	Heneicosane	296.344	000629-94-7	24.793	0.81 ± 0.22	n.i	n.i	24.793	1.10 ± 0.17	n.i	n.i
47	Linoleic acid	294.256	002566-97-4	n.i	n.i	n.i	n.i	24.729	± 0.03	25.106	2.22 ± 0.09
48	7,10,13-Hexadecatrienoic acid, methyl ester	264.209	056554-30-4	n.i	n.i	n.i	n.i	n.i	n.i	25.161	3.45 ± 1.12
49	Cyclododecyne	164.157	001129-90-4	25.313	0.56 ± 0.12	n.i	n.i	n.i	n.i	25.313	1.75 ± 0.25
50	Tricosane	324.376	000638-67-5	26.569	0.96 ± 0.08	n.i	n.i	26.573	1.25 ± 0.21	26.573	4.03 ± 1.25
51	1-(2,2-dimethyl-1-phenylethynylcyclopropyl)-Ethanol	214.136	1000268-53-7	n.i	n.i	n.i	n.i	n.i	n.i	26.641	5.31± 0.14
52	Acetic acid, 9,9-dioxo-9-thiabicyclo [3.3.1] non-6-en-2-yl ester	230.061	1000185-79-6	n.i	n.i	n.i	n.i	n.i	n.i	27.005	2.86 ± 0.89
53	Pentacosane	352.407	000629-99-2	28.206	0.34 ± 0.08	n.i	n.i	28.205	0.45 ± 0.22	28.206	4.56 ± 1.12
54	2-Hexenedioic acid, 2-methoxy-, dimethyl ester	202.084	056114-71-7	n.i	n.i	n.i	n.i	n.i	n.i	28.903	1.06 ± 0.95
55	l-Cysteine, *N*,*S*-bis (cyclohexylcarbonyl)-, methyl ester	355.182	1000282-52-4	n.i	n.i	n.i	n.i	n.i	n.i	29.254	1.26 ± 0.02
56	Hentriacontane	436.501	000630-04-6	n.i	n.i	n.i	n.i	n.i	n.i	29.719	1.10 ± 0.29
57	Eicosane	282.329	000112-95-8	n.i	n.i	n.i	n.i	n.i	n.i	31.132	0.73 ± 0.28
58	4-Trimethyl-3-hydroxymethyl-5a-(3-methyl-but-2-enyl)-cyclhexene	222.198	1000144-10-5	32.185	1.79 ± 0.74	n.i	n.i	n.i	n.i	n.i	n.i
59	Ursodeoxycholic acid	392.293	000128-13-2	n.i	n.i	n.i	n.i	n.i	n.i	35.504	0.55± 0.25
60	Chola-5,22-dien-3-ol, (3. β.,22*Z*)-	342.292	057597-14-5	n.i	n.i	n.i	n.i	n.i	n.i	35.656	1.82 ± 0.87

^a^ Relative content of individual compounds are expressed as average relative percent peak area after three replicated (*n* = 3), ^b^ RT-Retention times; ^c^%A—Peak areas, n.i. = not identified.

**Table 2:** Antioxidant activities of essential oils (EOs) extracted by different methods.

Samples	IC_50_ (mg/mL)	
	DPPH	ABTS
Ascorbic acid	0.08 ± 0.01^a^	0.12 ± 0.02 ^a^
HD	0.53 ± 0.03 ^c^	1.14 ± 0.08 ^c^
SD	0.58 ± 0.05 ^c^	0.84 ± 0.07 ^b^
SFME	0.43 ± 0.01 ^b^	0.75 ± 0.03 ^b^
SFE	0.42 ± 0.03 ^b^	1.05 ± 0.11 ^c^

^a–c^ Means in the same row followed by different letters are significantly different (*p* < 0.05). Each value is represented in terms of mean (*n* = 3) ± SD (Standard deviation).

**Table 3:** Minimal inhibitory concentrations (MIC) of essential oils extracted from *Flos Chrysanthemi indici* by different methods.

Method	MIC (µg/mL)					
	*X. oryzae*	*R. solanacearum*	*P. syringae*	*A. avenae*	*R. solani*	*E. carotovora*
Positive control	0.1	25.0	20.0	2.50	--	1.95
HD	62.5	125.0	62.5	62.5	125.0	125.0
SD	125.0	125.0	62.5	125.0	125.0	125.0
SFME	62.5	125.0	62.5	62.5	125.0	62.5
SFE	62.5	62.5	62.5	125	125.0	62.5
